# Active *Helicobacter pylori* Infection and Atrial Fibrillation in Patients Hospitalized With Acute Myocardial Infarction

**DOI:** 10.1111/anec.70245

**Published:** 2026-10-15

**Authors:** Mohammed Hashem, Martin O. Sundqvist, Jonatan Wärme, Magnus Bäck, Robin Hofmann

**Affiliations:** ^1^ Department of Medicine Karolinska Institutet, Solna Stockholm Sweden; ^2^ Department of Clinical Science and Education, Division of Cardiology Karolinska Institutet, Södersjukhuset Stockholm Sweden; ^3^ Department of Cardiology Karolinska University Hospital Stockholm Sweden

**Keywords:** acute myocardial infarction, atrial fibrillation, *Helicobacter pylori*, inflammation, urea breath test

## Abstract

**Objective:**

Chronic *Helicobacter pylori* ( *H. pylori* ) infection may promote systemic inflammation and has been proposed as a contributor to atrial fibrillation (AF), but clinical evidence is inconsistent. The aim of the study was to explore whether active *H. pylori* infection was associated with AF in patients hospitalized with acute myocardial infarction (AMI).

**Methods:**

This cross‐sectional analysis included adults from a two‐center observational AMI cohort. Active *H. pylori* infection was assessed during hospitalization using a urea breath test (UBT). AF was defined as AF documented before admission or identified during the index hospitalization. Associations were estimated using logistic regression adjusted for age, sex, center, and smoking. Exploratory multivariable interaction analyses were also performed.

**Results:**

Of 310 enrolled patients, 300 had conclusive *H. pylori* screening results; 60 (20.0%) were positive. AF was recorded in 4 of 60 *H. pylori* ‐positive patients (6.7%) and 30 of 240 *H. pylori* ‐negative patients (12.5%). *H. pylori* positivity was not significantly associated with recorded AF (unadjusted OR, 0.55; 95% CI, 0.17–1.41; *p* = 0.228; adjusted OR, 0.54; 95% CI, 0.16–1.46; *p* = 0.235). These estimates refer to pooled AF; only four AF cases occurred among patients testing positive.

**Conclusions:**

No statistically significant association was detected between *H. pylori* breath‐test positivity and pooled documented AF among patients hospitalized with AMI, but a clinically meaningful association cannot be excluded. Larger prospective studies with longitudinal follow‐up are needed to clarify whether active *H. pylori* infection is associated with incident AF after AMI.

## Graphical abstract

In a two‐center cohort of 300 patients hospitalized with acute myocardial infarction, active *Helicobacter pylori* infection was not significantly associated with atrial fibrillation prevalence (6.7% in infected versus 12.5% in uninfected patients).

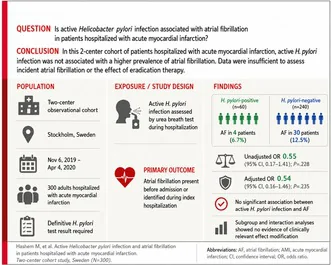

## 1 Introduction

Atrial fibrillation (AF) is the most common sustained cardiac arrhythmia and is associated with stroke, heart failure, hospitalization, and mortality. AF frequently complicates acute myocardial infarction (AMI), either as a pre‐existing comorbidity or as new‐onset AF triggered by myocardial ischemia, hemodynamic stress, and inflammation. Its reported prevalence in acute coronary syndrome ranges from 2% to 23%, depending on patient characteristics, infarct severity, monitoring, and diagnostic definitions (Van Gelder et al. 2024). New‐onset AF after AMI is not necessarily transient and has been associated with increased risks of ischemic stroke and mortality (Fauchier et al. 2021; Petersen et al. 2021). Concomitant AF also complicates antithrombotic management, as the need for stroke prevention must be balanced against the bleeding risk associated with combined anticoagulant and antiplatelet therapy (Van Gelder et al. 2024; Byrne et al. 2023).

The mechanisms underlying AF after AMI are multifactorial. Concurrent inflammation, oxidative stress, endothelial dysfunction, extracellular‐matrix turnover, and fibrosis are central components of atrial myopathy, creating a vulnerable substrate on which acute triggers can initiate AF (Tubeeckx et al. 2024; Nattel et al. 2020). These processes may amplify pre‐existing atrial vulnerability during AMI and support investigation of potentially modifiable inflammatory contributors. In this context, a biological link between *H. pylori* and AF is plausible, as chronic infection may promote several of these same inflammatory and vascular pathways and thereby contribute to atrial electrical and structural remodeling.

*H. pylori* is a gram‐negative, microaerophilic bacterium that chronically colonizes the gastric mucosa. Despite declining prevalence, infection remains common worldwide and varies substantially by geography and socioeconomic conditions (Li et al. 2023). Persistent infection causes chronic gastritis and is a major risk factor for peptic ulcer disease, gastric adenocarcinoma, and mucosa‐associated lymphoid‐tissue lymphoma (Malfertheiner et al. 2022). Beyond its gastrointestinal effects, *H. pylori* has been linked to cardiovascular disease through chronic inflammation, oxidative stress, endothelial dysfunction, platelet activation, altered lipid metabolism, and immune cross‐reactivity (Fang et al. 2019). Strains expressing cytotoxin‐associated gene A (CagA) may induce stronger inflammatory responses, although evidence for strain‐specific cardiovascular effects remains inconsistent (Schimke et al. 2010; Wang et al. 2025).

Interest in *H. pylori* after AMI has primarily focused on upper gastrointestinal bleeding, particularly because patients commonly receive dual antiplatelet therapy and those with AF may also require anticoagulation. In the cohort underlying the present study, AMI patients underwent bedside urea breath testing (UBT); active *H. pylori* infection was identified in approximately 20% of patients, and screening during hospitalization was feasible (Wärme et al. 2021). The subsequent HELP‐MI SWEDEHEART trial confirmed the feasibility of large‐scale screening but did not show a statistically significant reduction in the primary endpoint of upper gastrointestinal bleeding in an unselected AMI population, although the overall effect estimate favored screening and was compatible with a potentially clinically meaningful reduction. Notably, predefined subgroup analyses suggested greater benefit among patients at higher bleeding risk, particularly those with anemia and kidney failure, and a post hoc analysis suggested benefit among patients not discharged on proton pump inhibitor therapy (Hofmann et al. 2025). Neither study was designed to evaluate the association between active *H. pylori* infection and AF.

Small observational studies have reported associations between *H. pylori* , inflammatory markers, and AF, whereas later systematic reviews and cohort analyses have yielded inconsistent or null findings (Montenero et al. 2005; Wang et al. 2015; Yan et al. 2016; Tetta et al. 2019; Farah et al. 2024). Interpretation is complicated by confounding because *H. pylori* infection clusters with smoking, socioeconomic factors, and cardiometabolic disease, while AMI itself induces a substantial inflammatory response.

The aim of the study was to explore whether active *H. pylori* infection was associated with AF in patients hospitalized with AMI and to explore whether this association differed across clinically relevant patient subgroups, including age, sex, center, MI type, hypertension, diabetes, smoking, recent PPI use, and recent antibiotic use. These subgroup analyses were exploratory; pooled AF was not an incident‐AF endpoint.

## 2 Methods

### 2.1 Study Design

This was a cross‐sectional analysis of a two‐center observational cohort of adults hospitalized with AMI at Karolinska University Hospital Huddinge (H cohort) and Södersjukhuset (S cohort), Stockholm, Sweden, between November 6, 2019, and April 4, 2020, as previously described (Wärme et al. 2021). Eligible patients were aged ≥ 18 years; unwillingness or inability to provide informed consent was an exclusion criterion. The Swedish Ethical Review Authority approved the study (2019‐03846), all participants provided written informed consent, and the study was registered at ClinicalTrials.gov (NCT04289012). Clinical characteristics, treatments, and in‐hospital outcomes were obtained from electronic medical records and the Swedish Web System for Enhancement and Development of Evidence‐Based Care in Heart Disease Evaluated According to Recommended Therapies (SWEDEHEART). The AF analysis was secondary to the previously reported screening results. Enrollment overlapped with the first COVID‐19 wave in Stockholm; its potential effects on recruitment and testing were not assessed in this report.

### 2.2 *H. pylori* Status

Active *H. pylori* infection was assessed during the index hospitalization using a bedside UBT (Diabact, Mayoly Spindler) incorporated into routine in‐hospital AMI care (Wärme et al. 2021). *H. pylori* status was determined using the recommended cut‐off specified in the screening protocol. The primary exposure population was restricted to patients with a conclusive screening result ( *H. pylori* ‐positive or *H. pylori* ‐negative); inconclusive and otherwise non‐binary results were excluded. All *H. pylori* ‐positive patients were prescribed eradication therapy and referred to their general practitioner for follow‐up upon hospital discharge. PPI use during the preceding 2 weeks and antibiotic use during the preceding 4 weeks may suppress UBT positivity; a reported sensitivity analysis excluded patients with either recent exposure.

### 2.3 Outcomes

AF was defined as any documented AF present before admission or AF identified during the index AMI hospitalization. Each patient was counted once, regardless of whether AF was pre‐existing or detected during hospitalization. The available results do not separate pre‐existing from first‐detected AF, and first detection during admission does not establish onset after UBT. AF status was obtained from clinical records and the admission electrocardiogram. Uniform telemetry duration, number of additional electrocardiograms, minimum episode duration, and independent adjudication were not specified in the report.

### 2.4 Statistical Analysis

Continuous variables are presented as medians with interquartile ranges and categorical variables as *n*/*N* (%). Baseline characteristics are summarized descriptively without hypothesis‐testing *p*‐values. The primary association between *H. pylori* status and AF was estimated using logistic regression adjusted for age, sex, center, and smoking. This limited set represented demographic factors, recruitment center, and smoking while restricting model complexity in a cohort with 34 pooled AF cases; it was not a comprehensive adjustment for AF determinants, and no prespecified selection algorithm was reported. Unadjusted odds ratios (ORs), 95% confidence intervals (CIs), and *p*‐values were also calculated. Effect modification across clinically relevant subgroups was evaluated using adjusted multivariable logistic regression models containing *H. pylori* status, the candidate modifier, and an *H. pylori* ‐by‐modifier interaction term. Models were adjusted for age, sex, center, and smoking, except when one of these variables was the candidate modifier, in which case it was included only once as a main effect. An interaction ratio was calculated that describes how the *H. pylori* ‐associated OR changes across levels of the candidate modifier, while the *p*‐value for interaction tests whether this difference is statistically significant. Interaction effects were reported as ratios of *H. pylori* ‐associated ORs with 95% CIs, and *p*‐values for interaction were obtained using penalized likelihood‐ratio tests. Only nominal interaction *p* values are presented; multiplicity‐adjusted interaction *p* values are unavailable in the reported results, so these tests are descriptive and cannot establish effect modification or its absence. The sensitivity analysis excluded patients with PPI use in the preceding 2 weeks or antibiotic use in the preceding 4 weeks. A penalized coefficient estimator was not specified for the main logistic model; the penalized likelihood‐ratio tests described above apply to interactions. Analyses were performed using GraphPad Prism (Version 10.6.1). Tests were two‐sided, with *p* < 0.05 considered statistically significant.

## 3 Results

Of 310 enrolled patients, 10 were excluded because of inconclusive (*n* = 4), gray‐zone (*n* = 3), low‐CO_2_ (*n* = 1), or non‐binary *H. pylori* results (*n* = 2). The primary population therefore comprised 300 patients: 240 (80.0%) *H. pylori* ‐negative and 60 (20.0%) *H. pylori* ‐positive; 112 were from the H‐cohort and 188 from the S‐cohort (Figure 1). Median age was 67 years; 73 patients (24.3%) were women. Patients with AF were older than those without AF, with a median age of 78.0 years compared with 65.0 years. *H. pylori* positivity was less frequent among patients with AF than among those without AF (11.8% vs. 21.1%). Patients with AF more frequently had hypertension (82.4% vs. 60.5%), diabetes (35.3% vs. 25.9%), previous myocardial infarction (26.5% vs. 18.4%), recent PPI use (58.8% vs. 30.5%), and recent antibiotic use (17.6% vs. 8.7%). In contrast, ST‐elevation myocardial infarction and current smoking were less frequent among patients with AF (26.5% vs. 41.0% and 11.8% vs. 23.3%, respectively) (Table 1). Previous‐PCI status was reported for 282 of 300 participants, and information on recent antibiotic use was available for 299 of 300, as shown by the denominators in Table 1.

**FIGURE 1:**
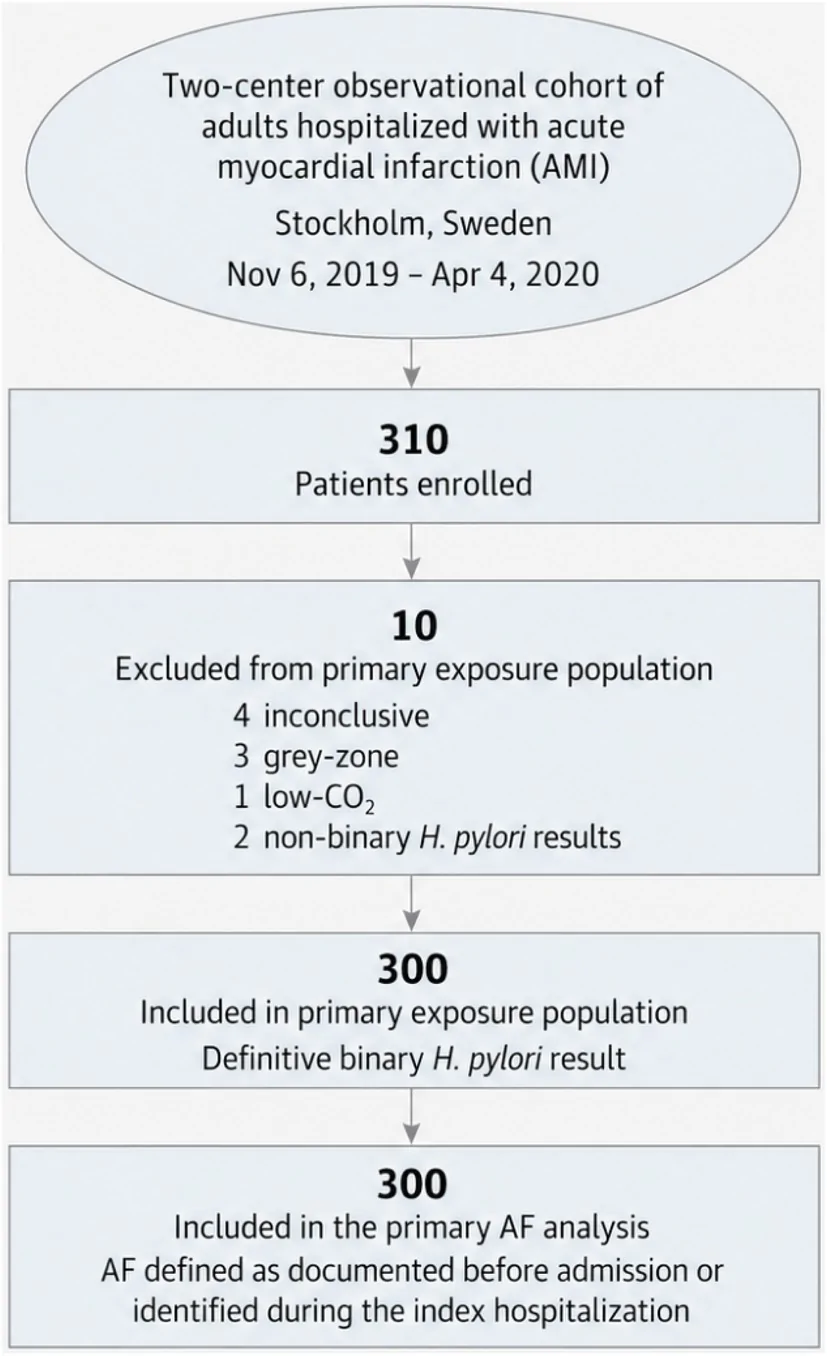
Study flow and analysis of populations. Flow diagram showing patient inclusion, exclusions from the *H. pylori* exposure population, and the primary AF analysis population. The primary analysis included patients with a conclusive *H. pylori* screening result. AF was defined as AF recorded before or during the index hospitalization. It does not represent an incident‐AF analysis population. AF, atrial fibrillation; *H. pylori* , *Helicobacter pylori* .

**TABLE 1:** Characteristics of the patients.

Characteristic	No. (%) of patients		
	Overall	No AF (*N* = 266)	AF (*N* = 34)
Demographics and cohort			
*H. pylori* ‐positive	60/300 (20.0%)	56/266 (21.1%)	4/34 (11.8%)
Age, years	67.0 (58.0, 76.0)	65.0 (57.0, 75.0)	78.0 (69.5, 82.0)
Female sex	73/300 (24.3%)	65/266 (24.4%)	8/34 (23.5%)
H‐cohort	112/300 (37.3%)	102/266 (38.3%)	10/34 (29.4%)
AMI presentation and risk factors			
ST‐elevation myocardial infarction	118/300 (39.3%)	109/266 (41.0%)	9/34 (26.5%)
Hypertension	189/300 (63.0%)	161/266 (60.5%)	28/34 (82.4%)
Diabetes	81/300 (27.0%)	69/266 (25.9%)	12/34 (35.3%)
Chronic kidney disease	31/300 (10.3%)	26/266 (9.8%)	5/34 (14.7%)
Previous myocardial infarction	58/300 (19.3%)	49/266 (18.4%)	9/34 (26.5%)
Previous stroke	17/300 (5.7%)	14/266 (5.3%)	3/34 (8.8%)
Previous PCI	54/282 (19.1%)	47/252 (18.7%)	7/30 (23.3%)
Smoking and recent medication exposure			
Current smoking	66/300 (22.0%)	62/266 (23.3%)	4/34 (11.8%)
PPI use during previous 2 weeks	101/300 (33.7%)	81/266 (30.5%)	20/34 (58.8%)
Antibiotic use during previous 4 weeks	29/299 (9.7%)	23/265 (8.7%)	6/34 (17.6%)

*Note:* Baseline characteristics are presented for the overall study population and according to whether AF was present. Continuous variables are reported as median (interquartile range), and categorical variables as *n*/*N* (%). Denominators vary where values are missing, including previous PCI (282/300) and recent antibiotic use (299/300); the AF groups combine previous and first‐detected AF.

Abbreviations: AF, atrial fibrillation; *H. pylori* , *Helicobacter pylori* ; PCI, percutaneous coronary intervention; PPI, proton pump inhibitor.

AF was recorded in 30 of 240 *H. pylori* ‐negative patients (12.5%) and 4 of 60 *H. pylori* ‐positive patients (6.7%), corresponding to an unadjusted OR of 0.55 (95% CI, 0.17–1.41; *p* = 0.228). After adjustment for age, sex, center and smoking, *H. pylori* positivity was not significantly associated with AF (OR, 0.54; 95% CI, 0.16–1.46; *p* = 0.235). When patients with recent PPI or antibiotic use were excluded, the association with AF remained non‐significant (OR, 0.79; 95% CI, 0.17–2.88; *p* = 0.733) (Figure 2). The medication‐restricted estimate is exploratory: its sample size, AF event counts, and baseline characteristics were not available in the reported output, so it cannot resolve possible differential false‐negative testing.

**FIGURE 2:**
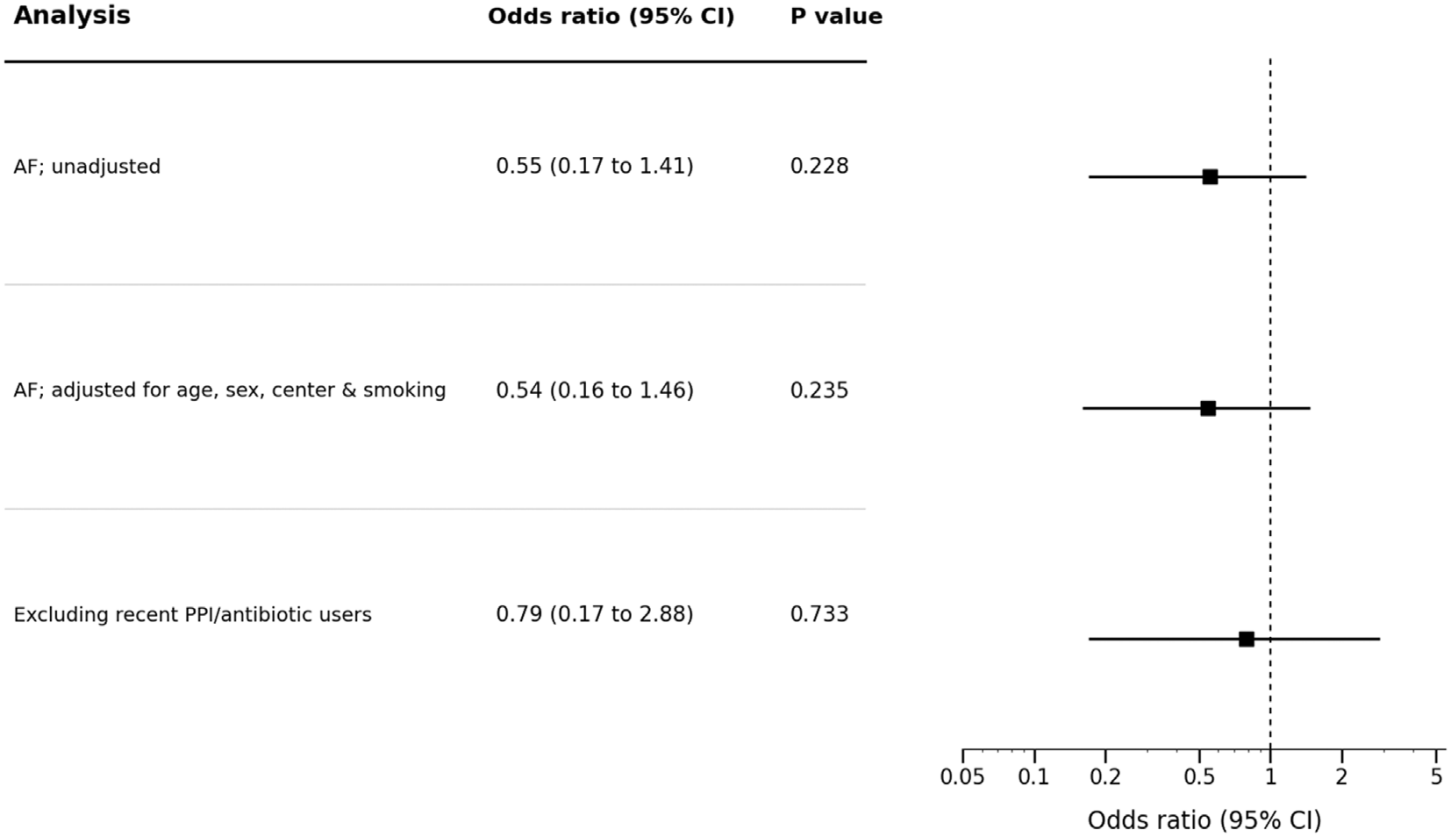
Association between *H. pylori* status and AF. Forest plot shows odds ratios comparing *H. pylori* ‐positive with *H. pylori* ‐negative patients across the primary analyses. Points represent odds ratios, horizontal lines represent 95% confidence intervals, and labels report the corresponding *p*‐values. These exploratory estimates concern pooled documented AF; the restricted subgroup denominator and event count were not reported. AF, atrial fibrillation; CI, confidence interval; *H. pylori* , *Helicobacter pylori* ; OR, odds ratio; PPI, proton pump inhibitor.

Fifteen exploratory adjusted multivariable interaction analyses are displayed in Figures 3 and 4. All nominal interaction *p*‐values were > 0.05, with the lowest observed for sex (*p*‐interaction = 0.177); with only four AF events in patients testing positive, these tests cannot reliably assess effect modification. Subgroup estimates were imprecise, with wide confidence intervals (Figures 3 and 4).

**FIGURE 3:**
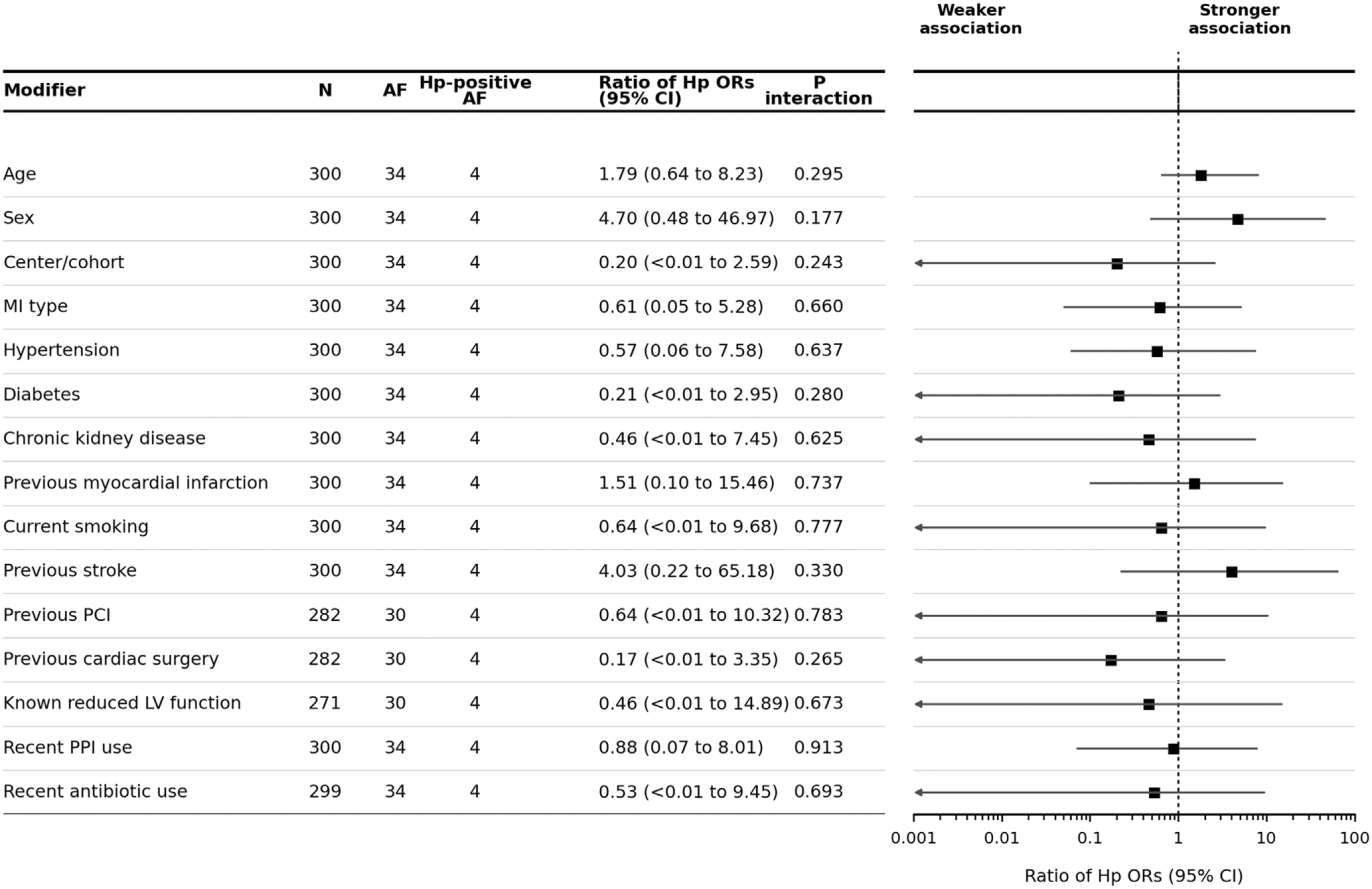
Adjusted multivariable analyses of *H. pylori* ‐by‐covariate interactions for AF. Ratios of *H. pylori* ‐associated odds ratios for the multivariable interaction analyses. Points represent interaction ratios, horizontal lines represent 95% confidence intervals, and the vertical dashed line at 1 indicates no interaction. Values below 1 indicate a weaker *H. pylori* ‐AF association at the higher or comparison level of the modifier, whereas values above 1 indicate a stronger association. All shown interaction *p*‐values are nominal; with four AF events among UBT‐positive patients, these plots cannot establish that subgroup effects are absent. AF, atrial fibrillation; *H. pylori* , *Helicobacter pylori* ; OR, odds ratio.

**FIGURE 4:**
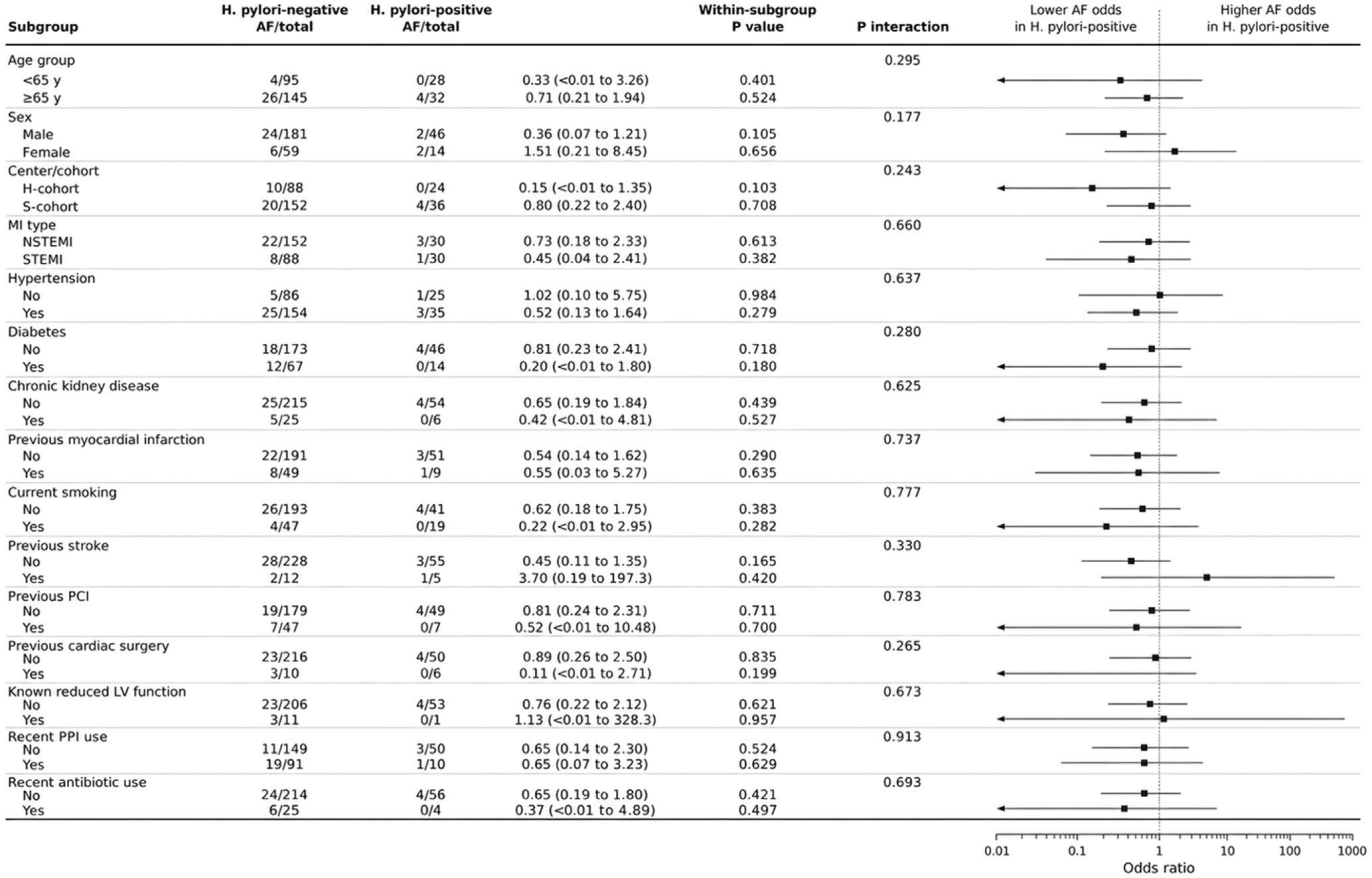
Adjusted association between *H. pylori* status and AF within clinically relevant subgroups. Points represent adjusted odds ratios comparing *H. pylori* ‐positive with *H. pylori* ‐negative patients within each subgroup; horizontal lines represent 95% confidence intervals, and the vertical dashed line at 1 indicates no association. Models were adjusted for age, sex, center, and smoking, with the corresponding adjustment term omitted when it defined the subgroup. Within‐subgroup *p*‐values assess the *H. pylori* –AF association within each subgroup. Values below 1 indicate lower AF odds and values above 1 indicate higher AF odds among *H. pylori* ‐positive patients. All subgroup estimates and nominal *p*‐values are exploratory; with only four AF events among UBT‐positive patients, they cannot assess effect modification reliably. AF, atrial fibrillation; CI, confidence interval; *H. pylori* , *Helicobacter pylori* ; LV, left ventricular; MI, myocardial infarction; NSTEMI, non‐ST‐elevation myocardial infarction; OR, odds ratio; PCI, percutaneous coronary intervention; PPI, proton pump inhibitor; STEMI, ST‐elevation myocardial infarction.

## 4 Discussion

In this two‐center study of patients hospitalized with AMI, we did not detect a statistically significant association between active *H. pylori* infection and pooled documented AF. The primary estimate was directed toward lower odds of AF among *H. pylori* ‐positive patients, but the confidence interval was wide and included the null. This should not be interpreted as evidence of a protective effect or as excluding a clinically meaningful association. The limited adjustment and pooled outcome cannot establish an independent contribution or address newly occurring AF.

The results are consistent with the mixed clinical literature. Early small case–control studies reported higher *H. pylori* seropositivity and inflammatory‐marker concentrations among patients with AF, particularly persistent or long‐standing AF (Montenero et al. 2005; Wang et al. 2015). A later meta‐analysis suggested an overall association between *H. pylori* infection and arrhythmias, with geographical variation (Yan et al. 2016). However, an AF‐specific meta‐analysis of six retrospective studies involving 2921 participants found no significant overall association and reported substantial heterogeneity and possible publication bias (Tetta et al. 2019). A more recent observational study again linked *H. pylori* infection, AF, age, and C‐reactive protein but could not establish causality (Farah et al. 2024). The present small, selected cohort cannot resolve the different findings in previous studies.

Recent mechanistic and epidemiological studies further illustrate the distinction between biological plausibility and clinical effect. A recent study showed that CagA promoted inflammatory and osteogenic responses, as well as calcification, in human coronary artery smooth‐muscle cells (Sundqvist et al. 2023). Although this supports a possible vascular effect of *H. pylori* , the model concerned coronary calcification rather than atrial electrophysiology or AF. Similarly, a Swedish population‐based nested case–control study found that neither *H. pylori* seropositivity nor CagA‐positive infection was independently associated with incident myocardial infarction after multivariable adjustment (Sundqvist et al. 2025). In a prospective cohort of 1061 patients with AMI, *H. pylori* ‐seropositive patients had higher C‐reactive protein concentrations and a distinct inflammatory biomarker profile, but *H. pylori* status was not associated with major adverse cardiovascular events or mortality (Sundqvist et al. 2026). Together, these studies suggest that *H. pylori* may influence systemic inflammation without necessarily producing a measurable independent increase in cardiovascular events, while the present study describes documented AF within a selected AMI cohort.

The present study used a UBT to assess active infection during AMI hospitalization; Wang et al. previously used a 13C UBT, and Farah et al. studied patients investigated using gastroscopy and/or breath testing (Wang et al. 2015; Farah et al. 2024). Serological testing may remain positive after spontaneous clearance or successful eradication and cannot reliably distinguish previous exposure from ongoing infection (Malfertheiner et al. 2022). Breath‐test interpretation depends on recent medication exposure, and these data cannot resolve discrepancies in earlier AF studies.

However, the association was sensitive to recent PPI or antibiotic use. These medications can suppress bacterial activity and cause false‐negative UBTs (Malfertheiner et al. 2022). The movement of the estimate toward no association after excluding recently exposed patients suggests that exposure misclassification may have contributed to the inverse direction of the primary estimate. Differences in medication use may also reflect age, comorbidity, or healthcare utilization. Recent PPI use was reported by 58.8% of patients with AF versus 30.5% without AF, and antibiotic use by 17.6% versus 8.7%; false‐negative UBT results could thus have been concentrated in the AF group. The restricted analysis did not report the subgroup denominator or event counts and cannot quantify or eliminate this bias.

Any effect of chronic gastric infection may also be difficult to detect during AMI. AF in this setting is plausibly driven by several strong acute mechanisms, including myocardial ischemia, atrial stretch, ventricular dysfunction, autonomic activation, electrolyte disturbance, and systemic inflammation (Tubeeckx et al. 2024; Nattel et al. 2020). Against this background, a modest contribution from *H. pylori* may be clinically difficult to separate from the dominant effects of the infarction itself.

The present study could not determine whether eradication therapy prevents AF. All *H. pylori* ‐positive patients were prescribed eradication therapy after the cross‐sectional ascertainment of *H. pylori* status and AF. The HELP‐MI SWEDEHEART trial showed that large‐scale *H. pylori* screening after AMI is feasible but did not significantly reduce upper gastrointestinal bleeding in an unselected AMI population, although the effect estimates suggested potentially protective signals in higher‐risk subgroups, including patients with anemia and chronic kidney disease. AF was not evaluated as an outcome in that trial (Hofmann et al. 2025). Whether *H. pylori* screening or eradication has any role in AF prevention therefore remains to be established.

This study has several limitations. The observational, cross‐sectional design and pooled pre‐existing and first‐detected AF prevent causal or incident‐AF inference; first detection during admission may have preceded UBT. The small number of *H. pylori* ‐positive patients and AF reduced statistical power and produced wide confidence intervals, particularly for subgroup and interaction analyses. AF ascertainment relied on clinical records and the admission electrocardiogram, so intermittent or asymptomatic AF may have been missed; telemetry duration, repeated‐ECG counts, a minimum episode duration, and independent adjudication were not specified. Recent PPI or antibiotic use was substantially more common among patients with AF and may have caused differential false‐negative UBT results. Information on CagA status, infection duration, treatment adherence, eradication success, peak troponin, Killip class, quantitative left ventricular ejection fraction, left atrial size, C‐reactive protein, and longitudinal inflammatory biomarkers was unavailable in this report. Hypertension, diabetes, renal disease, prior infarction, and other AF determinants were not included in the main model; residual confounding and selection on AMI hospitalization, including possible index‐event bias, remain. Enrollment extended into the first COVID‐19 wave in Stockholm, but effects on recruitment, case mix, or breath‐testing practice were not assessed. No post‐discharge AF follow‐up outcome was reported. The interaction analyses cannot establish absence of effect modification with four exposed AF cases. The two‐center Swedish population may limit generalizability. Sweden is a relatively low‐prevalence setting for *H. pylori* compared with many other countries, which may limit the applicability of these findings to populations where *H. pylori* infection is substantially more common (Agréus et al. 2016).

In summary, we did n*o*t detect a statistically significant association between UBT positivity and pooled documented AF after AMI, but these data cannot exclude a clinically meaningful association. As this was primarily a prevalence study, the available data were insufficient to assess incident AF or the effect of eradication therapy. Larger prospective cohorts with systematic rhythm monitoring and longitudinal follow‐up are needed to determine whether active *H. pylori* infection is associated with incident AF after AMI.

## Author Contributions

Conceptualization: M.H. and M.B. Investigation, project administration, resources, data curation, writing – review and editing: M.H., M.O.S., R.H., J.W., M.B. Writing – original draft, methodology, formal analysis, interpretation of results: M.H. Funding: M.B. and R.H. Supervision: M.B. and R.H. The authors have read and approved the final manuscript and accept responsibility for the integrity of the work. All the authors take responsibility for all aspects of reliability and freedom from bias of the data presented and their discussed interpretation.

## Funding

The study was funded by the Swedish Research Council (2019‐00414), The Swedish Heart Lung Foundation (2021‐0275, 2024‐0419), and the Region Stockholm (RS 2020‐0314, RS 2022‐0674). R.H. was supported by the Swedish Heart Lung Foundation (2021‐0273) and the Region Stockholm (RS 2021‐0933). M.B. was supported by grants from the Swedish Research Council (grant number 2023‐02652), Hjärt‐Lung Fonden (grant number 2024‐0697), Region Stockholm (grant number RS2023‐0859). The sources of funding had no role in design and conduct of the study; collection, management, analysis, and interpretation of the data; preparation, review, or approval of the manuscript; and decision to submit the manuscript for publication.

## Disclosure

During preparation of the revised manuscript, the authors used generative AI‐assisted tools for language editing, formatting, and reference organization. The authors reviewed and edited all AI‐assisted output and take full responsibility for the content, analyses, interpretation, and conclusions of the manuscript.

## Conflicts of Interest

R.H. reports institutional lectures and advisory board fees from MSD/Pfizer and AstraZeneca, unrelated to this study. Mayoly Spindler Laboratories generously supported the diagnostics in this study but had no role in: design and conduct of the study; collection, management, analysis, and interpretation of the data; preparation, review, or approval of the manuscript; and decision to submit the manuscript for publication.

## Data Availability

The data supporting the findings of this study are not publicly available. The study was reviewed and approved by the Swedish Ethical Review Authority for research and publication related to the specified aims. The data contain personal information and are therefore protected under the European Union General Data Protection Regulation and applicable Swedish confidentiality legislation. No de‐identified participant‐level dataset, reasonable‐request access procedure, or executable analysis code accompanies this report. The displayed aggregate data do not permit independent replication of the reported models.
